# Balance Control Method for Bipedal Wheel-Legged Robots Based on Friction Feedforward Linear Quadratic Regulator

**DOI:** 10.3390/s25041056

**Published:** 2025-02-10

**Authors:** Aimin Zhang, Renyi Zhou, Tie Zhang, Jingfu Zheng, Shouyan Chen

**Affiliations:** 1 GAC R&D Center, Guangzhou 511434, China; zhangaimin1@gacrnd.com; 2 School of Electromechanical Engineering, Guangdong University of Technology, Guangzhou 510006, China; 3 School of Mechanical and Automotive Engineering, South China University of Technology, Guangzhou 511442, China; merobot@scut.edu.cn (T.Z.); zjf1599158198@163.com (J.Z.); 4 School of Mechanical and Electrical Engineering, Guangzhou University, Guangzhou 510006, China; maxcsy@gzhu.edu.cn

**Keywords:** bipedal wheel-legged robots, balance control, LQR controller, Stribeck friction model, PSO algorithm

## Abstract

With advancements in mobile robot technology, wheel-legged robots have emerged as promising next-generation mobile solutions, reducing design costs and enhancing adaptability in unstructured environments. As underactuated systems, their balance control has become a prominent research focus. Despite there being numerous control approaches, challenges remain. Balance control methods for wheel-legged robots are influenced by hardware characteristics, such as motor friction, which can induce oscillations and hinder dynamic convergence. This paper presents a friction feedforward Linear Quadratic Regulator (LQR) balance control method. Specifically, a basic LQR controller is developed based on the dynamics model of the wheel-legged robot, and a Stribeck friction model is established to characterize motor friction. A constant-speed excitation trajectory is designed to gather data for friction identification, and the Particle Swarm Optimization (PSO) algorithm is applied to determine the optimal friction parameters. The identified friction model is subsequently incorporated as feedforward compensation for the LQR controller’s torque output, resulting in the proposed friction feedforward LQR balance control algorithm. The minimum standard deviation for friction identification is approximately 0.30, and the computed friction model values closely match the actual values, indicating effective and accurate identification results. Balance experiments demonstrate that under diverse conditions—such as flat ground, single-sided bridges, and disturbance scenarios—the convergence performance of the friction feedforward LQR algorithm markedly surpasses that of the baseline LQR, effectively reducing oscillations, accelerating convergence, and improving the robot’s stability and robustness.

## 1. Introduction

With the advancement of robotics technology, new varieties of mobile robots, such as legged robots, wheeled robots, and biomimetic robots, have become significant research areas both domestically and internationally, addressing varied application needs in diverse scenarios [1]. Recently, two-wheeled mobile robots have gained popularity due to their simple structure, high mobility, and low manufacturing costs, and they are frequently used in factories and warehouses for material handling and other tasks [2]. However, these environments are often designed primarily for human use and include narrow passageways, steps, and debris, presenting considerable challenges for traditional two-wheeled robots [3]. In contrast, robots with legged structures provide superior maneuverability and adaptability in complex and unstructured environments [4]. Therefore, integrating legged structures into two-wheeled robots to form wheel-legged robots represents a promising next generation of mobile robots. Nonetheless, as a new type of under-actuated mobile robot, wheel-legged robots demand significant improvements in balance control and stability control mechanisms. Hence, this research primarily focuses on designing a balance control algorithm for bipedal wheel-legged robots to enhance their stability and robustness.

Drawing on the extensive history and relative maturity of research on two-wheeled mobile robots, most balance and stability control methods for bipedal wheel-legged robots are adapted from those developed for two-wheeled systems [5]. Balance control techniques for two-wheeled robots mainly include full-state feedback controllers, Linear Quadratic Regulators (LQRs), sliding mode control, and nonlinear, higher-order controllers [6,7,8]. In two-wheeled robot balance motion control research, the motion control of bipedal wheel-legged robots is explored from different perspectives, including single-wheel dynamics, whole-body dynamics, and intelligent learning methods. The single-wheel dynamics approach treats the leg structure as a unified entity and concentrates on wheeled motion. For instance, Zhang Chao et al. designed a six-degree-of-freedom bipedal robot, the SR600, modeling it as a variable-structure wheeled inverted pendulum [9]. They established an inverted pendulum kinematic model based on center of gravity constraints and developed a PID balance control strategy, proving its feasibility through experiments. Fahad Raza et al. analyzed the motion stability of wheel-legged robots, utilizing the LQR control method to manage the robot’s balance, steering, and translational positioning, verifying the effectiveness of the LQR method in balance control through simulations [10]. Additionally, Adam Kollarčík used an LQR controller based on a linearized two-wheeled inverted pendulum model to control the wheels while employing two PD controllers to regulate the robot’s legs, confirming the performance of both the LQR and PD controllers on an actual robot [11].

Single-wheel dynamics simplifies the dynamics model by neglecting the influence of the upper body, which, while yielding satisfactory control results, does not fully exploit the advantages of the legs in achieving more complex motion control. To address this limitation, many researchers have explored robot motion control from a whole-body dynamics perspective. For example, Xin Yaxian et al. constructed an overall dynamics model by integrating torso dynamics, wheel-legged dynamics, and contact force constraints between the wheels and the ground, proposing a whole-body control framework validated through simulations [12]. Victor Klemm et al. introduced the compact wheeled biped robot Ascento and implemented a robust, model-based dynamic LQR controller, demonstrating its ability to autonomously jump up steps and recover from falls in various positions via experiments [13]. Additionally, they proposed a hierarchical whole-body controller based on a rigid body dynamics model for Ascento, deriving a closed-form expression for its dynamic motion loop and experimentally verifying that the controller enhances the system’s adaptability to rough terrain and increases robustness [14]. Zhang Yanheng et al. introduced a two-wheeled jumping robot combining wheel movement with jumping motion, using a four-bar mechanism of equal length to achieve balance control of the inverted pendulum model of the prototype [15]. Xin Songyan et al. presented a dynamic motion planning and control framework for wheeled biped robots, incorporating a rolling motion model for a cart-linear inverted pendulum, designing obstacle scenarios to validate the proposed hybrid motion generation method [16]. Wang Yu et al. proposed a whole-body motion planning method capable of simultaneously achieving torso posture and dynamic balance control tasks, controlling the wheeled biped robot in a virtual environment to track the speed and lateral yaw rate during turns, thereby validating the motion planning method’s effectiveness [17]. Finally, Chen Hua et al. integrated an LQR-based wheel controller with a task-space whole-body controller for other joints using an interference observer, verifying the scheme’s effectiveness through simulations [18].

The whole-body dynamics model facilitates the realization of more complex motions through a comprehensive approach; however, it introduces challenges related to model complexity and high computational load. Regardless of whether single dynamics or whole-body dynamics is employed, the accuracy of motion control entirely depends on the model, resulting in relatively low adaptability. To overcome this limitation, some researchers have sought to enhance model adaptability using intelligent learning approaches. For example, Qian Qingwen et al. proposed a dynamic balance control method based on adaptive machine learning, with simulation tests indicating that without adaptive machine learning, the robot is prone to losing balance [19]. The proposed dynamic balance control method effectively manages the dynamic balance of two-wheeled self-balancing pendulum robots. Fahad Raza et al. introduced an LQR with an L1 adaptive controller for the balance control of bipedal wheel-legged robots. The experimental results demonstrate that the hybrid controller can compensate for model uncertainties and external disturbances, outperforming the model-based LQR controller significantly [20]. Wang Shuai et al. proposed a nonlinear controller based on interconnected damping distribution to achieve control under more general conditions [21]. Experiments showed that the proposed nonlinear controller could maintain the robot’s balance even when starting from initial angles far from the balance point. Cui Leilei et al. employed reinforcement learning and adaptive dynamic programming to derive a learning-based adaptive optimal control scheme, where the controller learns from input state data collected from the robot’s trajectory [22]. Experiments validated the data-driven adaptive controller’s effectiveness in balancing wheel-legged robots. Zhuang Yulun et al. studied precise jump control for wheeled bipeds based on torque planning and energy consumption optimization, proposing a torque planning method using Bayesian optimization and successfully achieving height control in a real robot [23].

The methods discussed above each demonstrated advantages and achieved relatively favorable motion control results. However, they do not consider hardware characteristics like drive motor friction. Dai Fuquan et al. introduced a two-wheeled inverted pendulum robot design method that incorporates friction compensation, indicating friction in the drive mechanism as a crucial factor that affects the robot’s self-balancing performance [24]. Experimental results confirmed this approach’s effectiveness. Consequently, to reduce model complexity and mitigate drive motor friction’s impact on motion control, this paper proposes a balance motion control method that integrates the robot’s dynamics with a motor friction model to achieve stable balance control for bipedal wheel-legged robots. Recognizing that motor friction can lead to poor convergence and oscillations during low-speed movements, a Stribeck friction identification model is constructed based on the robot’s dynamics model, and a friction feedforward LQR balance control method is proposed.

The contributions of this paper are as follows:

1. In response to the challenges of traditional mobile robots in unstructured environments, this paper presents the design of a bipedal wheel-legged robot. To overcome balance and stability control challenges, an LQR balance control algorithm is developed based on a dynamics model. Recognizing the in-wheel motor friction’s significant impact on the dynamics model’s accuracy during low-speed movements, a Stribeck friction identification model is constructed. Building upon this friction model, a friction feedforward LQR balance control algorithm for bipedal wheel-legged robots is proposed to enhance the robot’s stability.
2. To optimize the in-wheel motor friction parameters, a uniform-speed motion trajectory is designed to collect identification data. Utilizing the nonlinear friction identification model and data sequence, a PSO algorithm is proposed to identify the optimal friction parameter set.
3. An experimental platform for the wheel-legged robot is constructed, integrating the bipedal wheel-legged robot with an IMU sensor system and an industrial control computer equipped with a real-time operating system. Experiments on motor friction identification and speed tracking validate the proposed particle swarm optimized in-wheel motor friction parameter set’s effectiveness. Comparative experiments further confirm that the proposed friction feedforward LQR balance control algorithm reduces steady-state oscillations and enhances the robot’s stability.

**Figure 1:**
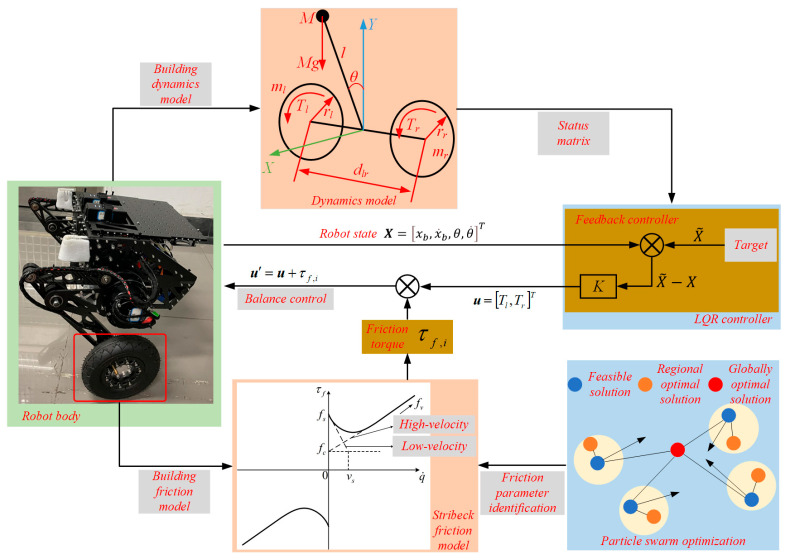
A structural diagram of the friction feedforward LQR Robot balance controller.

The overall structure of this paper is organized as follows: In Section 2, an LQR balance controller is developed based on the robot’s dynamics model, considering friction’s significant influence on robot motion during low-speed movements. A Stribeck friction identification model is established, introducing friction torque as feedforward compensation for the LQR output, and a friction feedforward LQR balance control algorithm is proposed for the robot. Section 3 identifies the optimal friction parameters by designing a uniform-speed motion trajectory to collect identification data sequences. Based on these data sequences and the friction model, a PSO algorithm is proposed to determine the optimal friction parameter set. Section 4 presents an experimental platform for the balance control of wheel-legged robots, and the proposed algorithm’s performance is analyzed in real-world balance control scenarios. Finally, Section 5 concludes this paper. The structural framework of the paper is illustrated in Figure 1.

## 2. Friction Feedforward LQR Balance Control Algorithm

### 2.1. LQR Balance Controller Based on Dynamics Model

**Figure 2:**
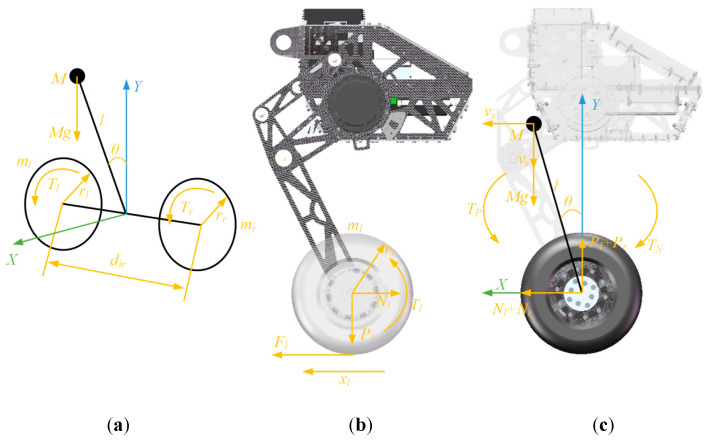
The equivalent model of the robot and a force analysis of each component: (**a**) the equivalent model; (**b**) the force analysis of the left drive wheel; (**c**) the force analysis of the chassis.

As illustrated in Figure 1, establishing a balance controller based on the robot’s dynamics model requires the construction of the dynamics model for the bipedal wheel-legged robot. For the purpose of balance control in this study, the following idealized assumptions are made regarding the robot: the chassis mass is considered equivalent to a concentrated mass located at the center of gravity, the influence of leg movement is neglected, and the drive wheels experience rolling friction without slipping against the ground [19]. Based on these assumptions, the balance control model of the wheel-legged robot can be effectively represented as a dual-wheel inverted pendulum model with a variable pole length. The entire model is divided into three components: the left drive wheel, the right drive wheel, and the frame. The equivalent model and force analysis are depicted in Figure 2.

Based on the force analysis shown in Figure 2b, the dynamic equation for the left drive wheel can be derived using Newton’s mechanics, expressed as follows:

(1) $${\ddot{x}}_{l}=\frac{T_{l}r_{l}-N_{l}{r_{l}}^{2}}{I_{l}+m_{l}{r_{l}}^{2}},$$

where $x_{l}$ denotes the displacement of the left drive wheel in the x-direction, $T_{l}$ is the driving torque applied to the left drive wheel, $r_{l}$ is the radius of the left drive wheel, $N_{l}$ is the horizontal reaction force exerted by the chassis on the left drive wheel, $I_{l}$ is the moment of inertia of the left drive wheel along its axis, and $m_{l}$ signifies the mass of the left drive wheel.

Similarly, since the force conditions for the right drive wheel are identical to those of the left drive wheel, the dynamic equation for the right drive wheel can be expressed as

(2) $${\ddot{x}}_{r}=\frac{T_{r}r_{r}-N_{r}{r_{r}}^{2}}{I_{r}+m_{r}{r_{r}}^{2}},$$

where $x_{r}$ denotes the displacement of the right drive wheel in the x-direction, $T_{r}$ is the driving torque applied to the right drive wheel, $r_{r}$ is the radius of the right drive wheel, $N_{r}$ is the horizontal reaction force from the chassis on the right drive wheel, $I_{r}$ is the moment of inertia of the right drive wheel along its axis, and $m_{r}$ is the mass of the right drive wheel.

At the same time, the acceleration of the chassis ${\ddot{x}}_{b}$ is the average of the axle accelerations of the left and right drive wheels. Given that the left and right drive wheels are identical, specifically $r_{l}=r_{r}$, $I_{l}=I_{r}$, and $m_{l}=m_{r}$, combining Equations (1) and (2) yields the following:

(3) $${\ddot{x}}_{b}=\frac{T_{l}r_{l}+T_{r}r_{l}-N_{l}{r_{l}}^{2}-N_{r}{r_{l}}^{2}}{2\left(I_{l}+m_{l}{r_{l}}^{2}\right)},$$

According to Figure 2c and Newton’s laws of motion, when conducting a force analysis with the center of mass of the chassis as the object of study, the dynamics equation for the chassis part can be expressed as

(4) $$\left(J_{z}+Ml^{2}\right)\ddot{\theta }=Mglsin\theta -Ml{\ddot{x}}_{b}cos\theta -T_{l}-T_{r},$$

where $J_{z}$ denotes the moment of inertia of the chassis along the z-axis, M is the equivalent mass of the chassis, l is the distance from the center of mass to the midpoint between the two drive wheels, and θ signifies the angle between the line connecting the center of mass and the centers of the two drive wheels and the vertical direction.

Furthermore, based on the force relationships between the chassis and the left and right drive wheels, the complete dynamic equation for the drive wheels can be derived by eliminating the forces described in Equation (3) as follows:

(5) $$\left(2I_{l}+2m{r_{l}}^{2}+M{r_{l}}^{2}\right){\ddot{x}}_{b}=T_{l}r_{l}+T_{r}r_{l}+M{r_{l}}^{2}l{\dot{\theta }}^{2}sin\theta -M{r_{l}}^{2}l\ddot{\theta }cos\theta ,$$

When the robot chassis experiences a small incline angle, indicating that it maintains balance within a limited angular range around the equilibrium position, the following linear approximations can be applied: cosθ=1, sinθ=θ, and ${\dot{\theta }}^{2}=0$. Consequently, after linearizing Equations (4) and (5), the dynamic equations governing the balance control of the wheel-legged robot can be derived as follows:

(6) $$\left\{\begin{array}{l} \left(J_{z}+Ml^{2}\right)\ddot{\theta }=Mgl\theta -Ml{\ddot{x}}_{b}-T_{l}-T_{r}\\ \left(2I_{l}+2m{r_{l}}^{2}+M{r_{l}}^{2}\right){\ddot{x}}_{b}=T_{l}r_{l}+T_{r}r_{r}-M{r_{l}}^{2}l\ddot{\theta } \end{array}\right.,$$

Based on the dynamics model established in Equation (6), the state space of the robot can be derived. By simplifying and combining terms of the same type, the robot’s state space is expressed as follows:

(7) $$\left\{\begin{array}{l} c_{1}{\ddot{x}}_{b}=T_{l}+T_{r}-c_{2}\ddot{\theta }\\ c_{3}\ddot{\theta }=c_{4}\theta -c_{5}{\ddot{x}}_{b}-T_{l}-T_{r} \end{array}\right.,$$

The coefficients in the equations satisfy the following expressions:

(8) $$\left\{\begin{array}{l} c_{1}=\left(2I_{l}+2m{r_{l}}^{2}+M{r_{l}}^{2}\right)/r_{l}\\ c_{2}=Mr_{l}l\\ c_{3}=J_{z}+Ml^{2}\\ c_{4}=Mgl\\ c_{5}=Ml \end{array}\right.,$$

Based on Figure 1, a balance motion controller can be constructed from the established dynamics model. However, to achieve improved motion control of the robot, it is crucial to select appropriate state variables and control variables. According to the motion control requirements outlined in this paper, the robot primarily aims to maintain point balance. To ensure optimal control performance, four variables are selected as state variables: the robot’s position, pitch angle, and their corresponding velocity variables. Thus, the state vector X can be expressed as follows:

(9) $$X={\left[x_{b},{\dot{x}}_{b},\theta ,\dot{\theta }\right]}^{T},$$

Control is achieved by regulating the torque of the two drive wheels, and the control vector u can be expressed as follows:

(10) $$u={\left[T_{l},T_{r}\right]}^{T},$$

Combining Equations (9) and (10), the state space representation is given by the following:

(11) $$\dot{X}=AX+Bu,$$

where $A\in R^{4\times 4}$ is a linearized dynamics matrix representing the relationships between the internal state variables of the system, and $B\in R^{4\times 2}$ is a control matrix that indicates the effect of the inputs on the various state variables.

Using the state space representation in Equation (11), the representation of Equation (7) can be transformed to match the state space form as follows:

(12) $$\left\{\begin{array}{c} {\ddot{x}}_{b}=\frac{-c_{2}c_{4}}{c_{1}c_{3}-c_{2}c_{5}}\theta +\frac{c_{2}+c_{3}}{c_{1}c_{3}-c_{2}c_{5}}\left(T_{l}+T_{r}\right)\\ \ddot{\theta }=\frac{c_{1}c_{4}}{c_{1}c_{3}-c_{2}c_{5}}\theta -\frac{c_{1}+c_{5}}{c_{1}c_{3}-c_{2}c_{5}}\left(T_{l}+T_{r}\right) \end{array}\right.,$$

In this case, matrices A and B are determined by the coefficients $c_{1}$, $c_{2}$, $c_{3}$, $c_{4}$, and $c_{5}$, which are fully defined based on the quality characteristics of the robot.

To implement closed-loop stability control for this unstable robotic system, a state feedback controller based on the state space must be designed. The LQR, a widely used full-state feedback controller in state space-based control scenarios [25], provides the control law.

(13) u=−KX,

By combining Equations (11) and (13), we obtain

(14) $$\dot{X}=\left(A-BK\right)X=A_{c}X,$$

where $K\in R^{2\times 4}$ is a feedback gain matrix.

To achieve a stable state for the wheel-legged robot system, the parameters of matrix K must be adjusted such that all eigenvalues of matrix $A_{c}$ are non-positive. To configure the poles for optimal control performance of the robot system, a target cost function J is introduced for the LQR robot balance controller, defined as follows:

(15) $$J=\frac{1}{2}\int_{0}^{\infty }\left(X^{T}QX+u^{T}Ru\right)dt,$$

where $Q\in R^{4\times 4}$ is a positive semi-definite matrix representing the penalty on the state variables, and $R\in R^{2\times 2}$ is a positive definite matrix representing the penalty on the control variable u. A larger component of matrix Q indicates faster convergence of the corresponding state variable to zero, while a larger component of matrix R corresponds to the magnitude of a particular control variable. Hence, following Equation (15), the optimization process entails adjusting matrices Q and R to minimize the cost function. Since matrices Q and R correspond to the state variable X and the control variable u, the objective is to adjust the state feedback controller in Equation (13) to minimize the cost function, ultimately reflected in the determination of the optimal feedback gain K. Substituting Equation (13) into (15) yields the following:

(16) $$J=\frac{1}{2}\int_{0}^{\infty }X^{T}\left(Q+K^{T}RK\right)Xdt,$$

It is assumed that there exists a symmetric positive semi-definite constant matrix P such that the following equation holds:

(17) $$\frac{d}{dt}\left(X^{T}PX\right)=-X^{T}\left(Q+K^{T}RK\right)X,$$

Differentiating the left side of Equation (17) yields

(18) $${\dot{X}}^{T}PX+X^{T}P\dot{X}=-X^{T}\left(Q+K^{T}RK\right)X,$$

Substituting Equation (14) into Equation (18) and rearranging them yields

(19) $$A^{T}P+PA+Q-K^{T}B^{T}P-PBK+K^{T}RK=0,$$

In Equation (19), matrices A, B, Q, R, and P are constant matrices, while K is the variable matrix. Therefore, the optimization problem translates to finding a matrix K that minimizes the cost function. By transforming the term containing matrix K into a structure similar to ${\left(M+N\right)}^{T}(M+N)$, minimizing the entire cost function occurs when M+N=0, from which matrix K can be derived.

Additionally, since R is a symmetric positive definite matrix, it is possible to find a displacement matrix such that $R=T^{T}T$ holds. Substituting this into Equation (19) yields

(20) $$A^{T}P+PA+Q-K^{T}B^{T}P-PBK+K^{T}T^{T}TK=0,$$

Transforming the term $-K^{T}B^{T}P-PBK+K^{T}T^{T}TK$ and using the method of undetermined coefficients to obtain the structure $M^{T}M+M^{T}N+N^{T}M+N^{T}N$ yields

(21) $$\left\{\begin{array}{l} M=-{\left(T^{-1}\right)}^{T}B^{T}P\\ N=TK \end{array}\right.,$$

Thus, the portion containing matrix K can be expressed as

(22) $$-K^{T}B^{T}P-PBK+K^{T}T^{T}TK={\left(M+N\right)}^{T}\left(M+N\right)-PBR^{-1}B^{T}P,$$

When M+N=0, it follows that

(23) $$TK-{\left(T^{-1}\right)}^{T}B^{T}P=0,$$

Solving Equation (23) yields

(24) $$K=R^{-1}B^{T}P,$$

Substituting Equation (24) into Equation (20) and simplifying it leads to

(25) $$A^{T}P+PA+Q-PBR^{-1}B^{T}P=0,$$

By selecting appropriate matrices Q and R to substitute into Equation (25), matrix P can be determined. The derived matrix P is then substituted into Equation (24) to calculate the optimal matrix K corresponding to matrices Q and R. Combining matrix K with the current state variables of the wheel-legged robot, the control vector for the next moment can be computed, thereby achieving the robot’s balance control. To enable the robot to track a trajectory, the reference input must also be included in the system input, as described by the following:

(26) $$u=K(\tilde{X}-X),$$

where $\tilde{X}$ is the robot’s desired state. In this study, $\tilde{X}$ is set to 0 to focus on balance control.

### 2.2. In-Wheel Motor Stribeck Friction Model

In Section 2.1, a dynamics-based LQR robot balance controller was constructed to control the robot by regulating the torque of the in-wheel motor. However, the dynamics model did not account for the effects of motor friction and internal resistance, which can hinder the robot’s ability to overcome friction at a low control torque, causing it to oscillate within a certain balance range. To enhance the performance of the LQR controller and optimize the robot’s convergence, friction compensation is introduced based on the LQR framework.

At high speeds, the torque output of the LQR controller is the primary factor, while the motor friction force becomes secondary. Conversely, at low speeds, the motor friction force emerges as the primary factor, with the nonlinear effects of friction, such as the Stribeck effect, playing a pivotal role. The Stribeck effect is characterized by a phenomenon where the friction force initially decreases and subsequently increases as the relative speed increases, reflecting the transition from static friction to Coulomb friction [26]. Thus, the Stribeck friction model can effectively describe the linear friction characteristics under high-speed conditions while accommodating the Stribeck effect and static friction effects during low-speed motion. According to the Stribeck principle [27], the classical Stribeck friction model can be expressed as follows:

(27) $$\tau_{f,i}=\left(f_{c,i}+\left(f_{s,i}-f_{c,i}\right)e^{-\left({\left|{\dot{q}}_{i}/v_{s,i}\right|}^{\xi_{i}}\right)}\right)\cdot sign\left({\dot{q}}_{i}\right)+f_{v,i}{\dot{q}}_{i},$$

In this equation, i=1,2 denotes the indices of the left and right in-wheel motors of the wheel-legged robot, $\tau_{f,i}$ is the friction torque, $f_{c,i}$ is the Coulomb friction coefficient, $f_{s,i}$ is the static friction coefficient, ${\dot{q}}_{i}$ is the motor speed, $v_{s,i}$ is the Stribeck speed coefficient, $\xi_{i}$ is the Stribeck curve decay coefficient, $sign\left(•\right)$ is the sign function, and $f_{v,i}$ is the viscous friction coefficient.

From Equation (27), it is evident that the classical friction model is based on the ideal assumption that the friction torque is completely symmetrical in both positive and negative velocity directions. However, practical factors such as manufacturing errors or wear of the motor structure can introduce deviations in the friction torque during forward and reverse motions. To better approximate the friction characteristics, a friction torque bias parameter is added to improve Equation (27):

(28) $$\tau_{f,i}=\left(f_{c,i}+\left(f_{s,i}-f_{c,i}\right)e^{-\left({\left|{\dot{q}}_{i}/v_{s,i}\right|}^{\xi_{i}}\right)}\right)\cdot sign\left({\dot{q}}_{i}\right)+f_{v,i}{\dot{q}}_{i}+f_{p,i},$$

where $f_{p,i}$ represents the friction torque bias.

From Equation (28), it can be observed that when the parameters $f_{c,i}$, $f_{s,i}$, $v_{s,i}$, $\xi_{i}$, $f_{v,i}$, and $f_{p,i}$ are known, the friction torque is solely dependent on the motor speed. Therefore, after calculating the friction torque based on speed, it can be utilized as feedforward compensation in the output torque of the LQR controller, thus enhancing the output of Equation (10):

(29) $$u^{\prime }=u+\tau_{f,i}={\left[T_{l},T_{r}\right]}^{T}+\tau_{f,i},$$

## 3. PSO for In-Wheel Motor Friction Parameter Identification Model

### 3.1. Establishing the Friction Identification Dataset

According to Equation (28), to identify the friction parameters $f_{c,i}$, $f_{s,i}$, $v_{s,i}$, $\xi_{i}$, $f_{v,i}$, and $f_{p,i}$, it is essential to obtain data pairs of motor speed and friction torque. During the robot’s operation, the motor speed and driving torque can be measured using encoders. However, the driving torque encompasses not only friction torque but also additional components, such as inertial torque [28], as illustrated by the following equation:

(30) $$\tau =M\left(q\right)\ddot{q}+C\left(q,\dot{q}\right)\dot{q}+G\left(q\right)+\tau_{f},$$

where τ represents the driving torque, $M\left(q\right)$ is the inertia, $C\left(q,\dot{q}\right)$ represents the Coriolis and centripetal forces, $G\left(q\right)$ is the gravitational force, and $\tau_{f}$ denotes the friction torque.

When acceleration is zero, the inertia term in Equation (30) vanishes. The term $C\left(q,\dot{q}\right)$, which represents velocity coupling between adjacent joints, can be disregarded since the in-wheel motor analyzed in this study operates independently. Furthermore, as the robot moves around the wheel’s center without the influence of gravity, the gravitational term $G\left(q\right)$ also becomes zero.

Therefore, while collecting data pairs for motor speed and friction torque, ensuring zero acceleration guarantees that the driving torque equals the friction torque. To acquire a more accurate identification dataset, a uniform motion excitation trajectory was designed to drive the motor. Consequently, Equation (30) can be simplified to

(31) $$\tau =\tau_{f},$$

During data collection, multiple gradient forward and reverse motion speeds were chosen to allow the in-wheel motor to operate along the predefined excitation trajectory, capturing speed and driving torque data throughout the control cycle. It is worth noting that the robot’s start and stop processes involve phases of acceleration and deceleration, which may introduce oscillations in the driving torque at the beginning and end. Therefore, data from the stable intermediate phases were utilized. The data collection process for determining the friction parameter set of the in-wheel motor is as follows:

1. Control the in-wheel motor *i* to move at a constant speed ${\dot{q}}_{i}$, collecting the motion speed ${\dot{q}}_{i}$ and driving torque $\tau_{i}$ at a specified frequency during movement.
2. Assume that 10 K data points are collected during motion, resulting in data sequences ${\left\{{\dot{q}}_{i}\left(k\right)\right\}}_{k=1}^{10K}$ and ${\left\{\tau_{i}\left(k\right)\right\}}_{k=1}^{10K}$.
3. Remove the first K data points and the last K data points. Divide the remaining eight K stable sequences into eight segments, computing the mean for each segment to obtain eight actual motion speeds ${\bar{\dot{q}}}_{i}$.
4. Perform the same operation on the driving torque to obtain the corresponding friction torque ${\bar{\tau }}_{f,i}$ for each speed ${\bar{\dot{q}}}_{i}$.
5. Select different motion speeds to generate excitation trajectories and repeat steps 1 to 4 to obtain the friction torques corresponding to different speeds.
6. Generate the speed and friction torque sequence ${\left\{{\left({\bar{\dot{q}}}_{i},{\bar{\tau }}_{f,i}\right)}_{n}\right\}}_{n=1}^{N}$, where N denotes the total number of data points.

Based on the set ${\left\{{\left({\bar{\dot{q}}}_{i},{\bar{\tau }}_{f,i}\right)}_{n}\right\}}_{n=1}^{N}$, the friction identification error for in-wheel motor *i* can be expressed as

(32) $$e_{i}=\frac{1}{N}\sum_{n=1}^{N}{\left({\bar{\tau }}_{f,i}\left(n\right)-{\hat{\tau }}_{f,i}\left(n\right)\right)}^{2},$$

where $e_{i}$ denotes the identification error for the *i*-th in-wheel motor, and ${\hat{\tau }}_{f,i}$ is the friction torque computed for the *i*-th motor, following the below equation:

(33) $${\hat{\tau }}_{f,i}\left(n\right)=\left(f_{c,i}+\left(f_{s,i}-f_{c,i}\right)e^{-\left({\left|{\bar{\dot{q}}}_{i}\left(n\right)/v_{s,i}\right|}^{\xi_{i}}\right)}\right)\cdot sign\left({\bar{\dot{q}}}_{i}\left(n\right)\right)+f_{v,i}{\bar{\dot{q}}}_{i}\left(n\right)+f_{p,i},$$

Thus, the friction parameter identification problem for the *i*-th in-wheel motor can be equivalently transformed into the following minimization optimization problem, which aims to identify a set of friction parameters that minimize the identification error within the feasible parameter range, represented as

(34) $$\underset{f_{c,i},f_{s,i},v_{s,i},\xi_{i},f_{v,i},f_{p,i}}{min}e_{i}\;s.t.\begin{array}{c} f_{c,i,min}<f_{c,i}<f_{c,i,max},f_{s,i,min}<f_{s,i}<f_{s,i,max}\\ v_{s,i,min}<v_{s,i}<v_{s,i,max},\xi_{i,min}<\xi_{i}<\xi_{i,max}\\ f_{v,i,min}<f_{v,i}<f_{v,i,max},f_{p,i,min}<f_{p,i}<f_{p,i,max} \end{array},$$

where the subscripts “*max*” and “*min*” denote the upper and lower bounds of the parameters, respectively.

### 3.2. Friction Parameter Identification Model Based on PSO

To comprehensively determine Equation (28) and subsequently establish Equation (29), thereby achieving balance and stability control of the wheel-legged robot based on the output of the proposed friction feedforward LQR algorithm, it is essential to find the optimal solution to problem (34). From Equation (28), it is evident that the established Stribeck friction model is nonlinear. The PSO algorithm, rooted in swarm intelligence and evolutionary computation, is particularly well suited for addressing nonlinear optimization problems, offering advantages such as rapid convergence, simple implementation, minimal parameter tuning, and effective performance in high-dimensional and complex optimization tasks [29]. By integrating PSO into the friction feedforward LQR algorithm, the stability control of the robot will be further enhanced.

In the PSO algorithm, a particle represents a candidate solution to an optimization problem. To address the friction parameters that need to be identified, we assume that the position of a particle corresponds to the set of friction parameters for in-wheel motor i, denoted as $x=[f_{c,i},f_{s,i},v_{s,i},\xi_{i},f_{v,i},f_{p,i}]$, where $x\in [x_{min},x_{max}]$ indicates the range of particle positions, with $x_{min}$ and $x_{max}$ representing the lower and upper limits, respectively. Additionally, each particle is assigned a velocity to determine the direction of its update. We assume the particle’s velocity to be $v=[v_{f_{c,i}},v_{f_{s,i}},v_{v_{s,i}},v_{\xi_{i}},v_{f_{v,i}},v_{f_{p,i}}]$, where $v\in [v_{min},v_{max}]$ indicates the range of velocities. Therefore, based on the principles of the particle swarm algorithm, the steps to find the optimal solution for the set of friction parameters of the in-wheel motor are depicted in the process outlined in Algorithm 1.
**Algorithm 1** Optimization process of particle swarm algorithm**Input:** Velocity and friction data pair sequence ${\left\{{\left({\bar{\dot{q}}}_{i},{\bar{\tau }}_{f,i}\right)}_{n}\right\}}_{n=1}^{N}$ **Output:** Optimal friction parameter set $f_{c,i},\;f_{s,i},\;v_{s,i},\;\xi_{i},\;f_{v,i},\;f_{p,i}$1.   Initialize iterations *t* = 0, maximum iterations *T*
2:   Initialize position permissible range *x_min_*, *x_max_*, Initialize velocity permissible range *v_min_*, *v_max_*
3:   **for** particle *i* = 1 to *N* **do**
4:       **for** dimension *d* = 1 to 6 **do**
5:         Initialize position *x_id_* and velocity *v_id_* randomly within permissible range
6:       **end for**
7:   Initialize particle optimal position with *x_i_*
8:   Calculate particle *i* fitness value
9:   **end for**
10:   Initialize global optimal position with the particle with the greatest fitness
11:   **for** *t* = 0 to *T*
**do**
12:       **for** particle *i* = 1, *N* **do**
13:         Calculate particle velocity according to $v_{n}^{t+1}=\omega v_{n}^{t}+c_{1}k_{1}\left(p_{n}^{t}-x_{n}^{t}\right)+c_{2}k_{2}\left(p_{g}^{t}-x_{n}^{t}\right)$
14:         Update particle position according to $x_{n}^{t+1}=x_{n}^{t}+\alpha v_{n}^{t+1}$
15:         Update inertia factor $\omega^{t}=\omega_{e}+\left(\omega_{s}-\omega_{e}\right)\left(T-t\right)/T$
16:         **if** position beyond permissible range **then**
17:           Restores position in last iteration, Inverse velocity, Update position according to new velocity
18:         **end if**
19:         Calculate particle *i* current fitness value
20:         **if** the fitness value is better than $p_{n}^{t}$ in history **then**
21:           Set current fitness value as the $p_{n}^{t+1}$
22:         **end if**
23:       **end for**
24:       **if** the optimal fitness value is better than optimal particle in history **then**
25:         Set optima fitness value as the $p_{g}^{t+1}$
26:       **end if**
27:       **if** the optimal fitness value is less than the threshold twice **break**
28:   **end for**
29:   Save optimal fitness value as the optimal friction parameter set result

According to Algorithm 1, the process for the PSO algorithm to find the optimal solution for the in-wheel motor’s friction parameter set is as follows:

1. Algorithm Initialization:

The iteration count (t=0) and the maximum iteration count T are set. The particle search space positions and velocity ranges are established as $\left[x_{min},x_{max}\right]$ and $\left[v_{min},v_{max}\right]$, respectively. N particles representing the friction parameter set within the specified range are randomly generated to form the initial population $X={\left\{x_{n}\right\}}_{n=1}^{N}$. The position of particle n is represented as

(35) $$x_{n}=[x_{n1},x_{n2},x_{n3},x_{n4},x_{n5},x_{n6}]=[f_{c,i},f_{s,i},v_{s,i},\xi_{i},f_{v,i},f_{p,i}],$$

Simultaneously, the velocity of particle n is represented as

(36) $$v_{n}=[v_{n1},v_{n2},v_{n3},v_{n4},v_{n5},v_{n6}]=[v_{f_{c,i}},v_{f_{s,i}},v_{v_{s,i}},v_{\xi_{i}},v_{f_{v,i}},v_{f_{p,i}}],$$

Thus, the individual optimal position of particle n is

(37) $$p_{n}=[p_{n1},p_{n2},p_{n3},p_{n4},p_{n5},p_{n6}],$$

The global optimal position of the entire population X is represented as

(38) $$p_{g}=[p_{g1},p_{g2},p_{g3},p_{g4},p_{g5},p_{g6}],$$

where n=1,2,⋯,N denotes the number of particles.

The particles within the position and velocity search ranges are randomly initialized as follows:

(39) $$x_{n}^{0}=x_{min}+k\left(x_{max}-x_{min}\right)\;v_{n}^{0}=v_{min}+k\left(v_{max}-v_{min}\right),$$

where k is a random number in the range of [0, 1]. The initial position of the particles is used to initialize the individual optimal positions, i.e., $p_{n}^{0}=x_{n}^{0}$.

The PSO algorithm utilizes fitness values to represent the search precision of each particle; higher fitness values indicate better solutions represented by the particles. According to the error representation of Equation (32), the reciprocal of the error is selected as the fitness function $F\left(x_{n}\right)$, meaning that smaller errors indicate higher search precision, resulting in larger corresponding fitness values, expressed as

(40) $$F\left(x_{n}\right)=\frac{1}{e_{i}\left(x_{n}\right)},n=1,2,\cdots N,$$

Next, the fitness values of the particles in the initialized population are calculated, and the particle with the highest fitness value is designated as the initial global optimal position.

2. Iterative Search for Optimal Parameter Solution:

The positions and velocities of all particles are iteratively updated:

(41) $$v_{n}^{t+1}=\omega v_{n}^{t}+c_{1}k_{1}\left(p_{n}^{t}-x_{n}^{t}\right)+c_{2}k_{2}\left(p_{g}^{t}-x_{n}^{t}\right)\;x_{n}^{t+1}=x_{n}^{t}+\alpha v_{n}^{t+1},$$

where ω is a non-negative inertia factor, $c_{1}$ and $c_{2}$ are non-negative learning factors, $k_{1}$ and $k_{2}$ are random numbers in the range of [0, 1], and α is a constraint factor. For particles that exceed the search range (i.e., $x_{n}^{t+1}<x_{min}$ or $x_{n}^{t+1}>x_{max}$), three adjustment steps are taken: the last iteration position $x_{n}^{t}=x_{n}^{t+1}-\alpha v_{n}^{t+1}$ is restored, the particle’s velocity $v_{n}^{t+1}=-v_{n}^{t+1}$ is inverted, and the particle’s position $x_{n}^{t+1}=x_{n}^{t}+\alpha v_{n}^{t+1}$ is re-updated.

The inertia factor ω is critical for the algorithm’s performance. In the initial stages, a larger inertia factor is utilized to enable the rapid convergence of particles near the optimal solution. In later stages, a smaller inertia factor allows particles to conduct more precise local searches around the current optimal solution. Consequently, the inertia factor decreases linearly with increasing iterations, expressed as

(42) $$\omega^{t}=\omega_{e}+\left(\omega_{s}-\omega_{e}\right)\frac{T-t}{T},$$

where $\omega_{s}$ and $\omega_{e}$ are the weights at the beginning and end of the iterations, respectively, and t and T represent the current and maximum iteration counts.

Next, the global optimum of the population and the individual optimal positions of the particles are updated. The fitness of particle n is calculated during the current iteration. If $F\left(x_{n}^{t+1}\right)>F\left(p_{n}^{t}\right)$, then the individual optimal solution of particle n to $p_{n}^{t+1}=x_{n}^{t+1}$ is updated; otherwise, $p_{n}^{t+1}=p_{n}^{t}$ is kept. Simultaneously, if $F\left(x_{n}^{t+1}\right)>F\left(p_{g}^{t}\right)$, the global optimal solution of the population to $p_{g}^{t+1}=x_{n}^{t+1}$ is updated; otherwise, $p_{g}^{t+1}=p_{g}^{t}$ is kept. The above operations are repeated until all particles have completed their updates.

3. Determination of Iteration Conditions:

The iteration process will stop when the current iteration number exceeds the set maximum iteration count T or when the fitness values of the global optimal solutions are both below a specified threshold in two consecutive iterations. In that case, the current global optimal position will be taken as the optimal solution for the friction parameters to be identified. Otherwise, step 2 should be followed to continue the iteration.

By utilizing the designed uniform excitation trajectory for data collection and applying the PSO algorithm offline, the friction parameters of the in-wheel motor can be effectively identified, thus enabling the accurate characterization of the Stribeck friction model. Substituting these identified parameters back into Equation (28) allows for the computation of friction force at a specific speed. This friction force is then fed forward as compensation in the LQR output torque according to Equation (29), facilitating the construction of a comprehensive friction feedforward LQR balance controller to achieve precise balance control of the robot.

## 4. Experiments and Analysis of Results

### 4.1. Experimental Platform for Balance Control of Bipedal Wheel-Legged Robot

**Figure 3:**
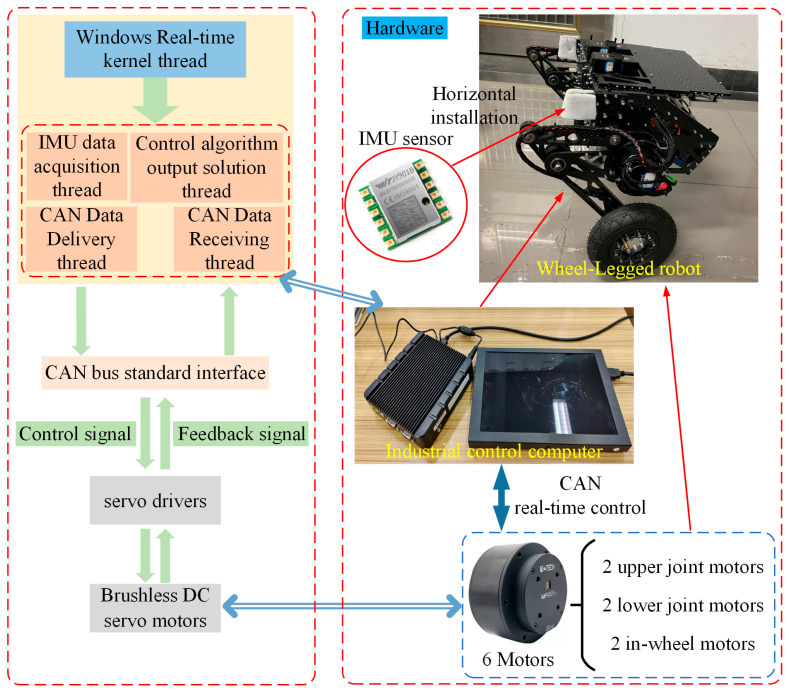
Experimental platform for balance control of bipedal wheel-legged robot.

To validate the proposed friction feedforward LQR balance control method, we designed a bipedal wheel-legged robot and constructed a hardware and software platform. The robot has a variable height ranging from 0.32 m to 0.42 m while maintaining a horizontal body orientation, with a mass of about 15 kg. This platform comprises the robot’s body, an IMU sensor module, an industrial control computer, and a real-time control module, with data collection and control at 1000 Hz. The self-designed robot supports wheel movement and leg posture adjustment. The IMU uses a JY901 nine-axis sensor for real-time orientation data at 100 Hz and a 0.01° resolution. The industrial control computer facilitates the robot’s real-time operation. Platform components are detailed in Figure 3. In balance control experiments, convergence is mainly determined by the in-wheel motors, with minimal influence from the joint motors. Motor details are shown in Table 1.

The real-time control module employs independent threads for tasks such as IMU data reception, motor data management via the CAN protocol, and motor control calculations. Thread communication is managed through global variables. The workflow includes the IMU thread parsing sensor data for motor control inputs; the motor data reception thread processing CAN data for control calculations; the control calculation thread integrating state variables to determine control outputs; and the motor data issuance thread converting and sending control signals to achieve a closed-loop control system.

### 4.2. Experiment and Analysis of Results for Stribeck Friction Parameter Identification Based on PSO

The Stribeck friction model established for the in-wheel motors of the wheel-legged robot in Section 2.2 comprises six parameters that need to be identified. To determine the parameter set and construct a complete friction model, the PSO algorithm developed in Section 3.2 is employed for the identification of these Stribeck friction parameters. As detailed in Section 3.1, an independent identification scheme for a single motor is utilized during the parameter identification process, and a uniform-speed motion trajectory serves as the identification trajectory. The motor speed is uniformly selected from the range of [−50, 50] rad/s, yielding a total of 260 data pairs that consist of motor speed and friction torque. The PSO algorithm is then applied to identify the parameter set based on these data. The key parameters for the PSO identification algorithm are shown in Table 2, and the upper and lower limits for the friction parameter set are provided in Table 3.

**Figure 4:**
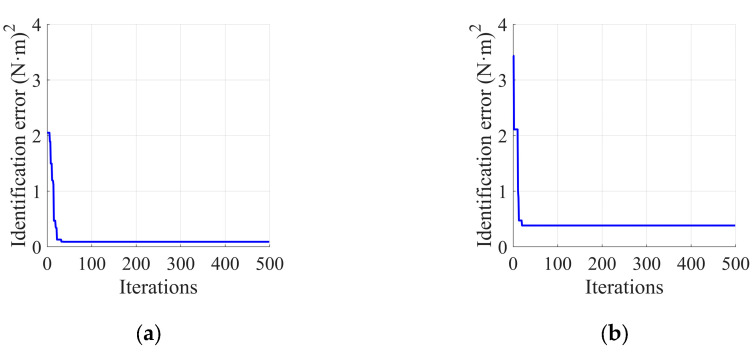
Iterative process of friction identification error: (**a**) left in-wheel motor; (**b**) right in-wheel motor.

**Figure 5:**
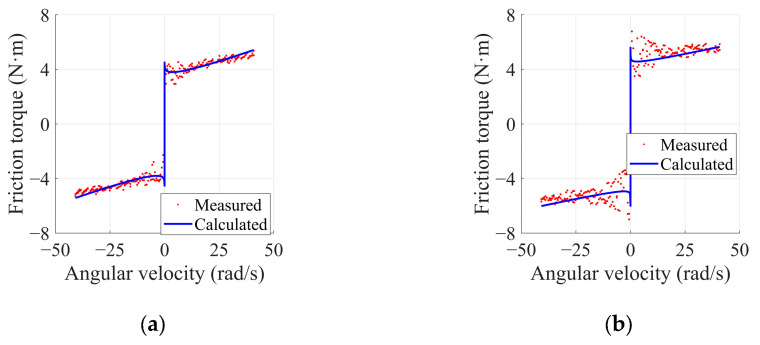
Fitting results of joint friction torque: (**a**) left in-wheel motor; (**b**) right in-wheel motor.

Based on the established parameters, friction identification was conducted on the left and right in-wheel motors. The iterative process of the friction identification error is illustrated in Figure 4, and the results of the Stribeck friction parameter set are presented in Table 4. The fitting results of the friction torque are depicted in Figure 5.

From Figure 4 and Table 3, the identification errors for both the left and right in-wheel motors decrease quickly, converging to their minimum values. Figure 5 shows that the calculated friction torque closely aligns with the measured torque, with the standard deviation of error being 0.30 and 0.59 for the left and right in-wheel motors, respectively, providing an initial validation of the PSO algorithm’s effectiveness in error identification.

### 4.3. Verification Experiment of Friction Identification Results Based on Speed Tracking and Analysis of Results

To further validate the effectiveness and accuracy of the friction identification results in Section 4.2, we conducted speed tracking experiments. The robot was positioned off the ground, with all other joints fixed, allowing only the wheels to be controlled for speed tracking. Two operational modes were established: one with friction torque compensation, and one without. The motors operated in torque control mode, using a PI feedback controller to track the target speed.

**Figure 6:**
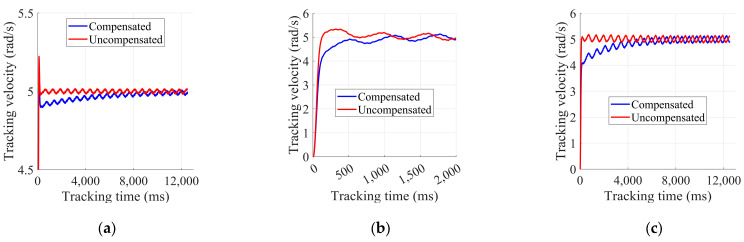
Results of left in-wheel motor speed tracking experiment: (**a**) kp = 0.5, ki = 0.05; (**b**) kp = 0.05, ki = 0.05; (**c**) kp = 0.05, ki = 0.01.

**Figure 7:**
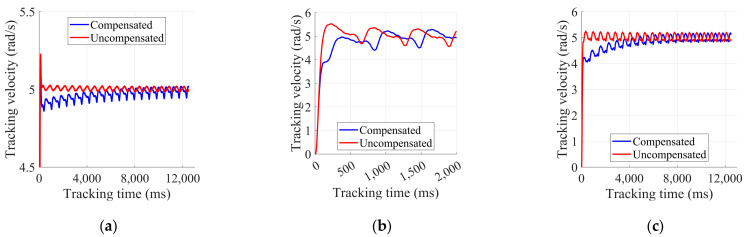
Results of right in-wheel motor speed tracking experiment: (**a**) kp = 0.5, ki = 0.05; (**b**) kp = 0.05, ki = 0.05; (**c**) kp = 0.05, ki = 0.01.

In the experiment, we set a target wheel speed of 5 rad/s for both operational modes, using different PI parameters. The speed tracking results for the left and right in-wheel motors are shown in Figure 6 and Figure 7. As illustrated, although the target speed could be tracked in both operational modes, the response was faster with friction compensation. Additionally, when the PI parameters were smaller, friction torque compensation enabled better tracking of the target speed. Smaller PID parameters can improve system stability and reduce oscillations and excessive responses, which are suitable for smoother control requirements. Compared to not using friction compensation, adding it enhanced the performance of the wheel controllers and improved the system’s control accuracy and dynamic responsiveness.

### 4.4. Experiments and Analysis of Results of Friction Feedforward LQR Balance Control

To validate the effectiveness of the FLQR balance control algorithm on a wheel-legged robot, we conducted a series of verification experiments. The original LQR algorithm served as a benchmark for comparison. To evaluate performance across different environments, experiments were performed under three conditions: flat ground, single-sided bridge, and disturbances. We analyzed changes in state variables, including displacement, pitch angle, and the speed of the robot. Notably, due to the IMU’s resolution being 0.01°, the pitch angle with the FLQR algorithm remained stable, close to a constant value rather than oscillating. The inability to stabilize at 0° was attributed to the installation error of the IMU and discrepancies between the theoretical and actual centers of mass.

1. Flat Ground Steady Point Balance Experiment

**Figure 8:**
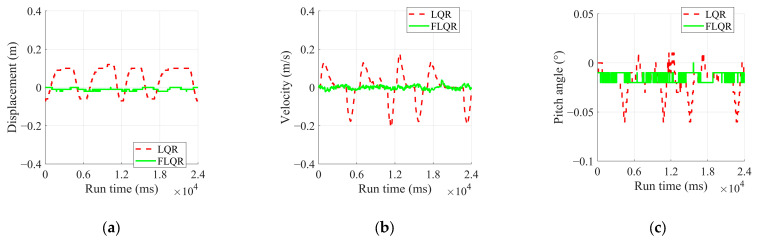
Experimental results of flat ground steady point balance experiment: (**a**) displacement; (**b**) velocity; (**c**) pitch angle.

**Figure 9:**
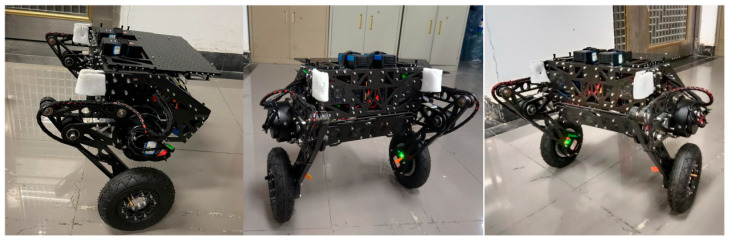
Process of flat ground steady point balance experiment.

The results of the flat ground steady point balance experiments are analyzed in Figure 8 and Table 5, while the experimental process is illustrated in Figure 9. From Figure 8, it can be observed that the displacement converges to −0.0092 m, with dynamic fluctuations ranging from −0.02 to 0 m, when using the FLQR balance control algorithm during experiments. The pitch angle converges to −0.015° with fluctuations between −0.02° and 0°. Since the robot is in a dynamic balance state, its speed fluctuates within a range of −0.026 to 0.038 m/s.

In contrast, the displacement in the two experiments using the LQR balance control algorithm does not show a convergence trend within a fluctuation range of −0.07 to 0.12 m. The pitch angle also lacks a convergence trend, fluctuating between −0.06° and 0.01°. Due to the larger displacement fluctuations, the robot’s speed varies widely between −0.21 and 0.18 m/s. Thus, compared to the LQR algorithm, the FLQR demonstrates a better convergence trend. The substantial dynamic fluctuations in the LQR balance algorithm are attributed to its inability to overcome frictional resistance as it approaches the steady state.

2. Single-Sided Bridge Steady Point Balance Experiment

**Figure 10:**
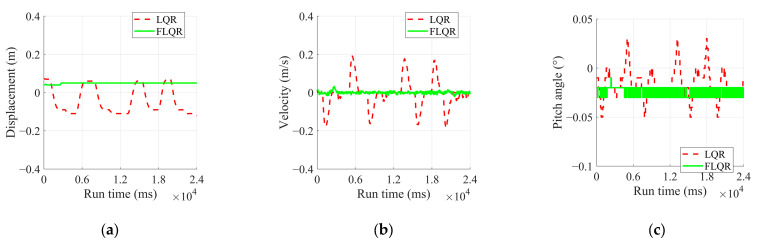
Experimental results of single-sided bridge steady point balance experiment: (**a**) displacement; (**b**) velocity; (**c**) pitch angle.

**Figure 11:**
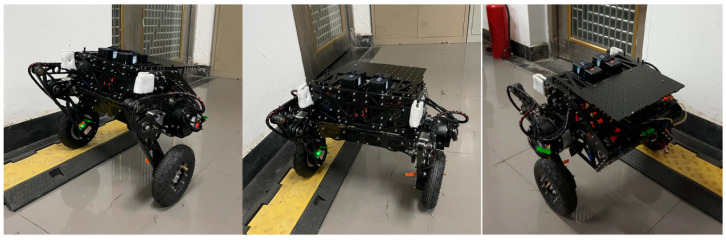
Process of single-sided bridge steady point balance experiment.

The results of the single-sided bridge steady point balance experiments are analyzed in Figure 10 and Table 6, while the experimental process is illustrated in Figure 11. From Figure 10, it can be observed that the results from the single-sided bridge experiments are similar to those from the flat ground steady point balance experiments, indicating that the robot maintains good stability in complex environments.

Specifically, for the single-sided bridge steady point balance experiments, the experiments performed using the FLQR balance control algorithm show displacements converging to 0.049 m within a fluctuation range of 0.03 to 0.05 m. The pitch angles converge to −0.02°, fluctuating between −0.03° and −0.01°. The speed fluctuates in the range of −0.0207 to 0.0324 m/s, consistent with the state observed during the flat ground steady point balance.

In contrast, the LQR balance control algorithm shows no convergence trend in displacement, with fluctuations remaining within ranges of −0.12 to 0.07 m. The pitch angle also lacks a convergence trend, fluctuating between −0.05° and 0.03°. The speed varies within the range of −0.18 to 0.19 m/s. Therefore, although the complexities of the single-sided bridge may introduce slight deviations in convergence positions across different trials, the FLQR algorithm still exhibits excellent convergence trends and stability, better meeting the requirements compared to the LQR algorithm.

3. Disturbance Rejection Experiment

**Figure 12:**
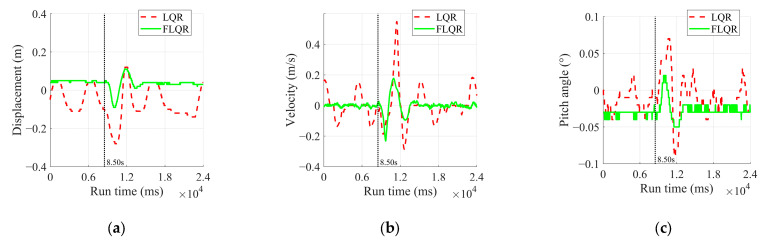
Experimental results of disturbance rejection experiment: (**a**) displacement; (**b**) velocity; (**c**) pitch angle.

**Figure 13:**
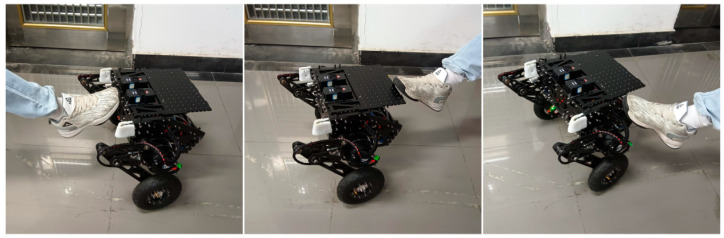
Process of disturbance rejection steady point balance experiment.

During the operation of both algorithms, the robot was kicked with the same force at different positions. An instance occurring at the 8.5 s mark of the robot’s operation was analyzed, and the results are presented in Figure 12 and Table 7. The specific experimental procedure is illustrated in Figure 13.

In Table 7, FLQR demonstrates superior control in the steady state by maintaining displacement tightly around 0.04 m, whereas LQR exhibits greater variability with displacements ranging from −0.1 to 0.04 m. Under disturbance conditions, FLQR keeps displacement within a narrow range of 0.09 to 0.11 m, while LQR shows a broader fluctuation between −0.28 and 0.11 m.

For velocity, FLQR effectively reduces oscillations, maintaining a steady-state range of −0.024 to 0.017 m/s. In contrast, LQR fluctuates more widely between −0.14 and 0.15 m/s. During disturbances, FLQR contains velocity variations ranging from −0.23 to 0.17 m/s, compared to LQR’s broader range from −0.29 to 0.55 m/s.

Regarding the pitch angle, FLQR maintains consistency with a steady-state value of approximately −0.03° and limits variations under disturbance to the range of −0.05° to −0.02°. In comparison, LQR shows more fluctuation, ranging from −0.04° to 0.03° in the steady state and from −0.09° to 0.07° under disturbance. These results confirm that FLQR offers improved disturbance rejection, ensuring tighter control of the position, velocity, and pitch angle during balance maintenance.

## 5. Conclusions

To enhance the adaptability of mobile robots in unstructured environments, a bipedal wheel-legged robot was designed. To reduce control model complexity and minimize the impact of drive motor friction on motion control, a friction feedforward LQR (FLQR) balance control method was developed, integrating the robot’s dynamics with a motor friction model to achieve stable balance control. The key findings of this paper are as follows:

1. A bipedal wheel-legged robot platform, comprising the robot itself, an IMU sensor, and an industrial control computer with a real-time system, was developed to improve adaptability in unstructured environments. To enhance balance and stability, an LQR balance control algorithm was constructed based on the dynamics model. In-wheel motor friction, which significantly affects model accuracy during low-speed movements, needs to be compensated due to its tendency to cause poor convergence and oscillations. A Stribeck friction identification model for the motor was established, and an FLQR balance control algorithm was proposed to improve the robot’s stability.
2. To optimize in-wheel motor friction parameters, a uniform-speed excitation trajectory was designed to collect data for friction identification. Using the nonlinear friction model and data sequences, a Particle Swarm Optimization (PSO) algorithm was employed to determine optimal friction parameters. The minimum standard deviation for friction identification is approximately 0.30, with the computed friction model values closely matching the actual values. The calculated friction torque aligns well with the measured torque. Without friction compensation, the motor could not track the target speed, whereas with friction compensation, the motor successfully tracked the target speed, validating the accuracy and effectiveness of the identification results.
3. To validate the effectiveness of the proposed FLQR balance control algorithm in reducing steady-state oscillations and enhancing stability, balance experiments were conducted on flat ground, a single-sided bridge, and under disturbance conditions. The results indicate that the FLQR algorithm achieves effective convergence across these scenarios, with steady-state variance in displacement, velocity, and pitch angle being reduced by at least one order of magnitude compared to the LQR. Additionally, under identical external disturbances, FLQR exhibited a significantly lower disturbance range than LQR, demonstrating its effectiveness in mitigating steady-state oscillations and improving the robot’s robustness.

## Figures and Tables

**Table 1:** Details of the motors.

Motors	Rated Torque (*N* ⋅ *m*)	Rated Speed (RPM)	Power (W)	Control Frequency (Hz)
Upper Joint Motors (HaiTai HT8115-J9)	20	120	160	1000
Lower Joint Motoes (HaiTai H8115-J36)	54	30	160	1000
In-wheel Motors (LKMTECH MF9025v2)	2.42	490	170	1000

**Table 2:** Parameter set for PSO algorithm.

Parameter	Particle Swarm Size (M)	Learning Factor (c1/c2)	Inertia Factor Upper/Lower Limits	Constraint Factor (*α*)	Velocity Limit	Number of Iterations
Value	128	1.6/2.0	0.9/0.4	0.6	0.5	500

**Table 3:** Upper and lower limits for Stribeck friction parameter set.

Parameter	$f_{c}\left(N\cdot m\right)$	$f_{s}\left(N\cdot m\right)$	$f_{v}\left(N\cdot m\right)$	$v_{s}\left(rad/s\right)$	ξ	$f_{p}\left(N\cdot m\right)$
Upper	3	10	10	10	2.2	10
Lower	0.01	0.01	0.01	0	0	−10

**Table 4:** Identified Stribeck friction parameter set results based on PSO algorithm.

Joint i	$f_{c}\left(N\cdot m\right)$	$f_{s}\left(N\cdot m\right)$	$f_{v}\left(N\cdot m\right)$	$v_{s}\left(rad/s\right)$	ξ	$f_{p}\left(N\cdot m\right)$
Left Motor	0.0338	0.0761	4.9 × 10^−4^	1.612	0.2310	−0.0016
Right Motor	0.0406	0.0617	4.3 × 10^−4^	2.253	0.2772	0.0014

**Table 5:** Analysis of results of flat ground steady point balance experiment.

Type	Method	Range	Mean	Variance
Displacement (m)	LQR	−0.07~0.12	0.0467	0.0645
	FLQR	−0.02~0	−0.0092	0.0068
Velocity (m/s)	LQR	−0.2066~0.1751	0	0.07
	FLQR	−0.0263~0.0384	0	0.0093
Pitch angle (°)	LQR	−0.06~0.01	−0.0188	0.0144
	FLQR	−0.02~0	−0.0147	0.005

**Table 6:** Analysis of results of single-sided bridge steady point balance experiment.

Type	Method	Range	Mean	Variance
Displacement (m)	LQR	−0.12~0.07	−0.0436	0.0692
	FLQR	0.03~0.05	0.049	0.003
Velocity (m/s)	LQR	−0.1797~0.1907	−0.0080	0.0759
	FLQR	−0.0207~0.0324	0	0.0061
Pitch angle (°)	LQR	−0.05~0.03	−0.0158	0.0152
	FLQR	−0.03~−0.01	−0.0243	0.005

**Table 7:** Analysis of results of disturbance rejection experiment.

Type	Method	Range or Value of Steady State	Range of Disturbance State
Displacement (m)	LQR	−0.1~0.04	−0.28~0.11
	FLQR	0.04	0.09~0.11
Velocity (m/s)	LQR	−0.14~0.15	−0.29~0.55
	FLQR	−0.024~0.017	−0.23~0.17
Pitch angle (°)	LQR	−0.04~0.03	−0.09~0.07
	FLQR	−0.03	−0.05~−0.02

## Data Availability

All data are contained within the article.
